# Clinical trials in neuromodulatory treatment of drug-resistant hypertension and the need for spinal cord stimulation trials: a PRISMA systematic review

**DOI:** 10.1186/s42234-024-00160-7

**Published:** 2024-12-02

**Authors:** Garrett W. Thrash, Elijah Wang, Yifei Sun, Harrison C. Walker, Prasad Shirvalkar, Bryan K. Becker, Marshall T. Holland

**Affiliations:** 1 Heersink School of Medicine, University of Alabama at Birmingham, 5663 Post Oak Trl, Bessemer, Birmingham, AL 35022 USA; 2 Department of Neurosurgery, University of Alabama at Birmingham, Birmingham, AL USA; 3 Department of Neurology, University of Alabama at Birmingham, Birmingham, AL USA; 4 Departments of Anesthesiology (Pain Medicine), Neurological Surgery and Neurology, University of California San Francisco, San Francisco, CA USA; 5 Department of Medicine, Division of Nephrology, University of Alabama at Birmingham, Birmingham, AL USA

**Keywords:** Neuromodulation, Spinal Cord Stimulation, SCS, Drug resistant Hypertension, Hypertension, Renal Denervation, Carotid Body Stimulation, Neurosurgery

## Abstract

**Background:**

Drug-resistant hypertension affects approximately 9–18% of the United States hypertensive population. Recognized as hypertension that is resistant to three or more medications, drug-resistant hypertension can lead to fatal sequelae, such as heart failure, aortic dissection, and other vast systemic disease. The disruption of the homeostatic mechanisms that stabilize blood pressure can be treated procedurally when medication fails. These procedures include carotid body stimulation, renal denervation, sympathectomies, dorsal root ganglia stimulation, and more recently spinal cord stimulation and have all been utilized in the treatment of drug-resistant hypertension.

**Methods:**

To identify the clinical trials of neuromodulation in drug-resistant hypertension, a PubMed search was performed that included all original clinical trials of neuromodulation treating drug-resistant hypertension. The 838 articles found were sorted using Covidence to find 33 unique primary clinical trials. There were no methods used to assess risk of bias as a meta-analysis was not feasible due to heterogeneity.

**Results:**

Renal denervation and carotid body stimulation have both shown promising results with multiple clinical trials, while sympathectomies have mostly been retired due to the irreversible adverse effects caused. Dorsal root ganglion stimulation showed varying success rates. Spinal cord stimulation is a novel treatment of drug-resistant hypertension that shows promising initial results but requires further investigation and prospective studies of the treatment to provide guidelines for future DRH treatment. The limitations of the review are reporting bias and absence of a meta-analysis that compares the treatment modality due to the heterogeneity of reported outcomes.

**Conclusion:**

Innovation in neuromodulation is necessary to provide alternative avenues of treatment in the face of contraindications for standard treatment. Treatment of drug-resistant hypertension is essential to delay dangerous sequelae. This review’s objective is to summarize the clinical trials for treatment of drug-resistant hypertension following PRISMA guidelines and suggests future directions in the treatment of drug-resistant hypertension.

## Background

Drug-resistant hypertension (DRH) is a major cause of heart disease, stroke, and chronic kidney disease (Avery et al. 2017; Fengler et al. 2016a; 2016b). Approximately 9–18% of the hypertensive patients have DRH defined as (1) hypertension resistant to treatment from three different classes of antihypertensives or (2) hypertension controlled with four or more medications (Doroszko et al. 2016). Blood pressure (BP) is regulated by the autonomic nervous system with the sympathetic nervous system playing an important role in increasing vascular tone, stroke volume, and heart rate (Charkoudian et al. 2009). The ANS receives feedback via baroreflex mechanisms primarily from the aortic and carotid sinus and carotid body baroreceptors signaling to the nucleus tractus solitaris in the brainstem (Schmieder 2016). An increase in BP increases the firing of the baroreceptors which results in multi-system peripheral effects, such as decrease in sympathetic tone, decrease in BP, and feedback activation of the renin–angiotensin–aldosterone system (Papademetriou et al. 2011a; 2011b; Zhang et al. 2014). The inhibition of the renin–angiotensin–aldosterone system can reduce reabsorption of water and sodium through actions of aldosterone and antidiuretic hormone in the kidney to decrease stroke volume and cardiac output while vasodilation decreases systemic vascular resistance (Van Beusecum et al. 2015). The heart also releases atrial natriuretic peptide and brain natriuretic peptide in response to the high pressure in the atria and ventricles caused by HTN (Katzman et al. 1990). This combined response normally functions to lower systemic BP in the absence of intervening pathology such as DRH.

Pathological disruption of autonomic nervous system homeostasis can lead to HTN. First line therapy is medication based. These including cardio-selective beta blockers, calcium channel blockers, angiotensin converting enzyme inhibitors, and angiotensin receptor blockers, among others (Hamdidouche et al. 2019; Lambertucci et al. 2011; Lang et al. 1992; Lang et al. 1993). However, in many refractory cases, other avenues of treatment are deployed that utilize neuromodulation to modify BP homeostasis signaling pathways. Neuromodulation is the process of regulating neuronal activity through altering the electrical activity of neural pathways through either stimulation or inhibition. Current neuromodulation treatments include renal denervation, carotid body stimulation, spinal cord stimulation, and dorsal root ganglion stimulation. Sympathectomies were previously utilized for DRH treatment but are no longer used due to the irreversible side effects associated with the procedure.

Electrical neural stimulation therapies include deep brain stimulation, intracranial cortical stimulation, renal denervation, dorsal root ganglion stimulation, and spinal cord stimulation (Johnson et al. 2013). Neuromodulation of the sympathetic nervous system of the kidney can be used to control DRH. Neuromodulation possesses capabilities to inhibit or stimulate certain neural structures—renal nerves, carotid body, DRG, and the spinal cord—to influence the autonomic nervous system (McCorry 2007). The neuromodulatory treatments this review covers treat DRH through peripheral stimulation or denervation of the sympathetic nervous system. The majority of these treatments inhibit the sympathetic nervous system and reduce its influence on the vascular system, but one of the treatments, known as renal denervation, irreversibly damages the renal nerves to remove sympathetic vascular tone. This systematic review follows PRISMA guidelines to summarize the findings of the most recent clinical trials in neuromodulatory treatment of DRH.

## Methods

We performed a systematic review of the literature on 10/26/2023 in PubMed using PRISMA guidelines included in the supplement for the following search terms:

(“ANS” [tiab] OR “Autonomic Nervous System” [tiab] OR “Autonomic Nervous System”[MeSH] OR “Neuromodulation” [tiab] OR “Renal Denervation” [tiab] OR “Carotid Body Stimulation” [tiab] OR “ Sympathectomy” [tiab] OR “Spinal Cord Stimulation” [tiab]) AND (“Kidney” [MeSH] OR “Renal hypertension” [tiab] OR “Hypertension” [MeSH]) AND (Clinical Trial “controlled clinical trial” [Publication Type] OR “clinical trial” [tiab]) (Welch et al. 2012).

**Fig. 1:**
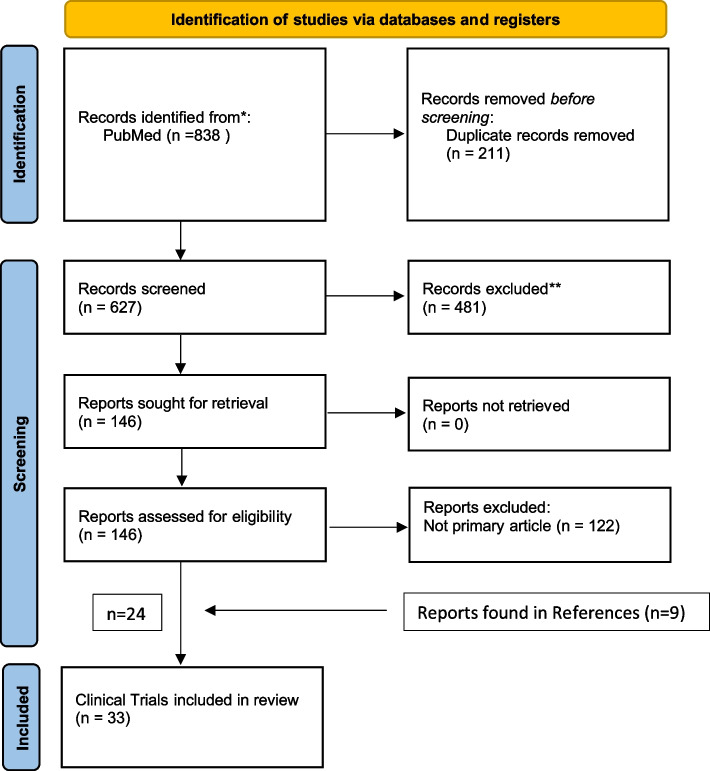
PRISMA flow diagram of the PubMed literature search

A total of 838 articles were exported and were screened independently by authors G.T. and E.W. using Covidence. All conflicts were resolved by M.H. Inclusion criteria were 1) original article of unique clinical trials that involved 2) neuromodulation therapy of DRH. All reviews, commentaries, and other reports on the clinical trials were excluded. We found 24 primary unique clinical trials for the treatment of DRH and reported them in this review. We then included 9 more trials found in the reference section for a total of 33 trials. The data was not systematically collected; however, a summary of each trial was synthesized by E.W. The review protocol was not registered in a database (Fig. 1).

## Main text

### Drug resistant hypertension

The cause of primary HTN is unknown, yet there are many risk factors associated with this disease. Some risk factors of primary HTN include, obesity, alcohol consumption, smoking, and excess sodium intake (Jordan et al. 1999). Secondary hypertension is less common and due to secondary sympathetic activation through causes such as pheochromocytoma, Cushing’s syndrome, thyroid or parathyroid dysfunction, obstructive sleep apnea, renal stenosis and more (Sarwar et al. 2013). While secondary HTN can be treated by addressing the offending agent, primary HTN requires lifelong medical intervention. Without treatment, patients are at high risk for a multitude of cardiovascular and systemic diseases, such as heart failure and aortic dissection (Ranard et al. 2019).

### Autonomic nervous system – physiology and targets to intervene/modulate

**Fig. 2:**
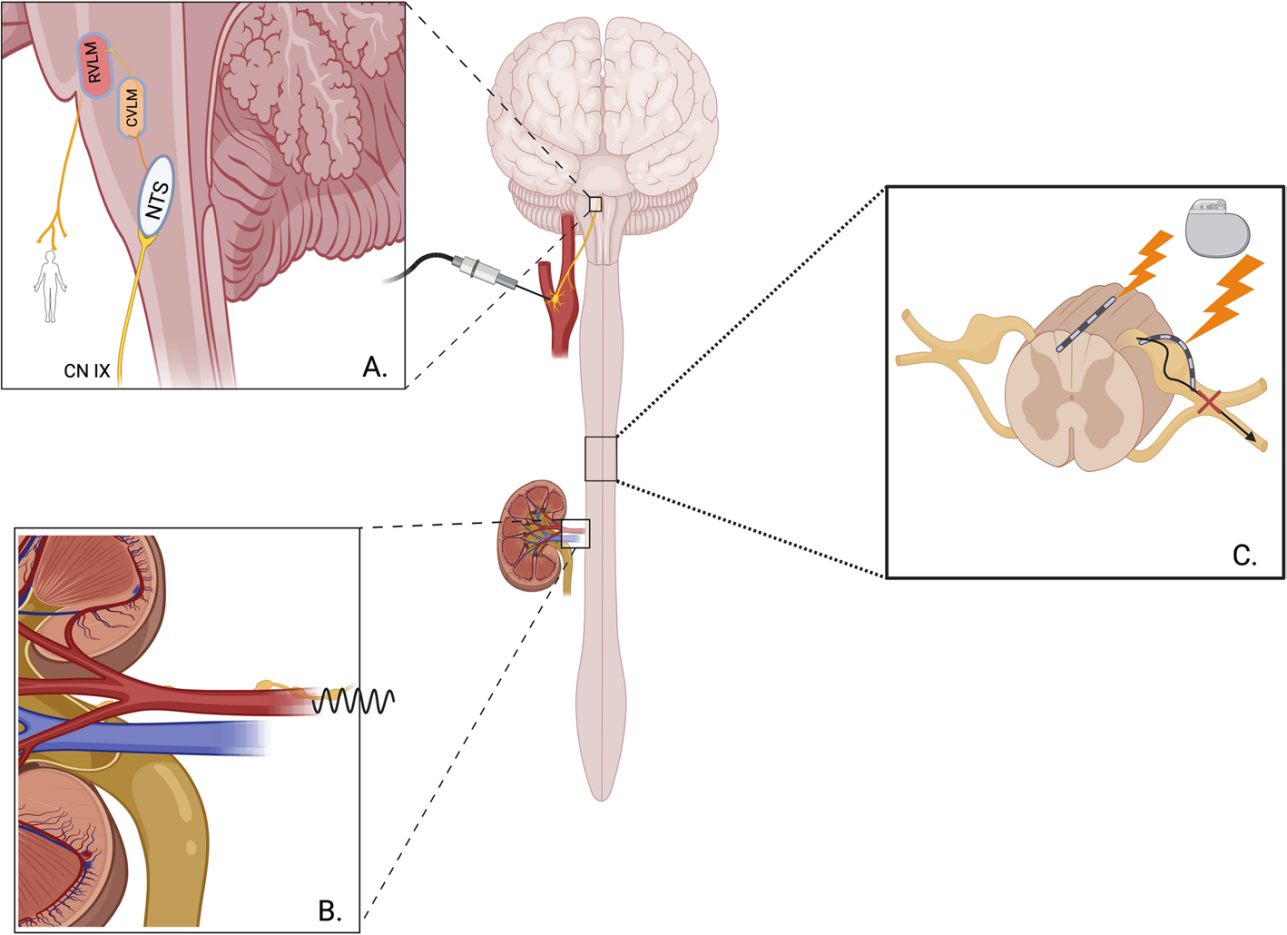
Conceptualization of neuromodulation modalities of drug-resistant hypertension treatment. **A** Effects of Carotid Body Stimulation: The carotid body is procedurally stimulated within the carotid sinus to simulate high mean arterial pressure. The afferent glossopharyngeal nerve travels up the nucleus tractus solitarius (NTS) into the caudal and rostral ventral lateral medulla (CVLM, RVLM) to inhibit sympathetic fibers. The inhibition of efferent sympathetic fibers lower the sympathetic vascular tone. **B** Renal Denervation: By removing the nerves along the renal artery, renal denervation prevents the adrenergic sympathetic fibers from acting upon the kidney’s many feedback mechanisms that can cause an increase in mean arterial pressure, such as the renin–angiotensin–aldosterone feedback system. **C** Spinal Cord and Dorsal Root Ganglion Stimulation: While the mechanism behind spinal cord stimulation requires further elucidation, it is believed that the procedure lowers mean arteriole pressure through decreasing postganglionic sympathetic nervous system activity

The autonomic nervous system, divided into the sympathetic and parasympathetic divisions, is responsible for maintaining various physiological homeostatic mechanisms, one of which is BP regulation (McCorry 2007). Imbalance of homeostasis leads to pathological sequelae, such as HTN. The mechanism of autonomic nervous system autoregulation of BP can be seen in Fig.2. The clinical trials mentioned throughout this review modulate the autonomic nervous system to restore the homeostatic balance in the event of HTN pathophysiology.

The aortic and carotid bodies are peripheral baroreceptors that provide constant feedback of the physiological state of the BP (Herlitz et al. 1993). The carotid body responds to both increases and decreases in mean arterial pressure. An increase in mean arterial pressure leads to a decrease of sympathetic firing through the glossopharyngeal nerve, which sends the signal to the nucleus tractus solitarius. The nucleus tractus solitarius decreases sympathetic tone leading to a decrease in systemic vascular resistance, and in turn mean arterial pressure. The aortic bodies respond to an increase in mean arterial pressure and signal through the vagus nerve, to the nucleus tractus solitarius for the same response (Sarwar et al. 2013). Mechanical stimulation of vagus and glossopharyngeal nerves is interpreted by the nucleus tractus solitarius as a persistent increase in mean arterial pressure which results in decreased sympathetic response through vasodilation and mean arterial pressure reduction (Sarwar et. al 2013).

While the main renal regulation of blood pressure lies in the tuberoglomerular and myogenic stretching feedback loops of the afferent and efferent renal arterioles through renin–angiotensin–aldosterone-system, the renal nerves also impact the ability of the kidneys to lower mean arterial pressure. The renal nerves respond to both mechanical and chemical stimuli that stimulate afferent nerve fibers to signal that the mean arterial pressure has increased (Hosseini-Dastgerdi et al. 2022). The efferent fibers from postsynaptic sympathetic ganglia to the renal arterioles are mostly adrenergic fibers that cause sodium and water retention, renal arteriole vasoconstriction, and renin–angiotensin–aldosterone-system activation of juxtaglomerular cells in response to norepinephrine release (Converse et al. 1992; Hausberg et al. 2007). This increase of sympathetic nervous system activity can be moderated through renal denervation therapies.

Neuromodulation of BP in the spinal cord has was also targeted in techniques such as sympathectomies (Beglaibter et al. 2002; Collin et al. 1994), DRG stimulation (Sverrisdottir et al. 2020), and spinal cord stimulation (Memar et al. 2023a; 2023b). The physiological mechanisms of each of these treatments are visualized in Fig. 2 and will be further discussed in their respective roles (Fig. 2).

### Carotid baroreceptor stimulation

The carotid baroreceptors are located in the carotid sinuses and are stretch-sensitive mechanoreceptors that relay signals through the glossopharyngeal nerve to the nucleus tractus solitarius where the afferent signals from the baroreceptors are received (Papademetriou et al. 2011a; 2011b). They function to balance sympathetic and vagal tone (Zhang et al. 2014). From the nucleus tractus solitarius, the signals received by the baroreceptors are sent to the caudal ventrolateral medulla where they are converted from excitatory signals to inhibitory signals that are then sent to the right ventrolateral medulla where sympathetic neurons travel throughout the body to modulate sympathetic tone (Papademetriou et al. 2011a; 2011b). In carotid body stimulation, the carotid baroreceptors are activated which decreases the sympathetic tone and causes systemic vasodilation (Papademetriou et al. 2011a; 2011b). This mechanism was first observed in 1965 by Bilgutay and Lillehei in dog models of HTN (Zhang et al. 2014).

Baroreflex activation therapy involves surgical subcutaneous implantation of a pulse generator in the infraclavicular chest wall pocket. The carotid sinus is approached via a transverse cervical incision above the carotid bifurcation for electrode placement, then the carotid sinus region is found through trial electrode placement and electrical stimulation in various areas to pinpoint the area most receptive area for therapy and permanent implantation (Gronda et al. 2017). Successful baroreceptor stimulation will result in the signal produced from the carotid sinus and aortic arch receptors arriving at the medulla oblongata’s nervous centers via afferent fibers that innervate the nucleus tractus solitarius (Lohmeier et al. 2019; Voora et al. 2018). This signal is interpreted by the brain as an increase in BP (Zhang et al. 2014). Inhibition in the rostral ventrolateral medulla occurs through complex neural interactions and results in a reduction in sympathetic tone to the heart, blood vessels, adrenal glands, kidneys, lungs, and other essential organs (Gronda et al. 2017). The therapy results in reduced adrenergic tone due to artificial stimulation to the alpha-adrenergic receptors that modulate arteriolar resistance and venous capacitance (Reid 1986). A decrease in BP is accompanied by a decrease in heart rate and muscle sympathetic nerve activity (Zhang et al. 2014). Long-term control of body fluid balance, thus BP, regulated by the kidney is also a result of carotid body stimulation. Drawbacks of carotid body stimulation include dependence on an external programming device for the pulse generator, the fact that it is unsuitable for certain types of HTN such as angiotensin II-induced HTN, and the fact that medication treatment is still required for carotid body stimulation to be effective; a case report showed BP rises when stimulation was conducted without antihypertensive medications, and the signature decrease in BP is only present when antihypertensive medications are used in tandem with carotid body stimulation.

### Carotid baroreceptor trials

Carotid baroreflex stimulation, or baroreflex activation therapy, lowers BP by using electricity to stimulate the carotid baroreceptors in the carotid sinus to decrease sympathetic outflow, induce vasodilation, and decrease mean arterial pressure.

The DEBuT-HT study was a 3 month study that included 45 patients utilizing the Rheos Baroreflex Hypertension Therapy Device by CVRx to combat DRH through baroreflex activation therapy (Scheffers et al. 2010). Eligibility criteria included SBP greater than 160 mmHg and/or a DBP greater than 90 mmHg while being treated with at least 3 antihypertensive medications at maximally tolerated doses with one of them being a diuretic (Heusser et al. 2010). The Rheos Baroreflex Hypertension Therapy Device by CVRx acted similar to a pacemaker by creating a pulse from a single pulse generator that had 2 electrode leads and 2 field electrodes attached to it that were subcutaneously tunneled into and attached to each carotid sinus (Bisognano et al. 2011). The pulse generator was placed in a subcutaneous pocket under the pectoralis with the leads tunneled subcutaneously upwards towards the bifurcation of the carotid artery to the carotid bulbs (Hoppe et al. 2012; Alnima et al. 2012; van Kleef et al. 2022). The leads’ positions were determined through intraoperative electrode testing to determine where they would provide the most therapeutic effects transcutaneously (Heusser et al. 2010; Bisognano et al. 2011). The electricity emitted from the device could have potentially interrupted wound healing, so investigators waited 1 month post-implantation to activate stimultion. After 3 months, the results of the DEBuT-HT study were an average SBP drop of 32 ± 10 mmHg. Upon turning the device off in clinic, the reduced BP returned to its previous hypertensive state at 193 ± 9/94 ± 5 mmHg. Chronic 24-h SBP and DBP decreased 10 ± 12 mmHg and 6 ± 10 mmHg respectively. The trial illustrated the safety and efficacy of baroreflex activation therapy using the Rheos Baroreflex Hypertension Therapy Device through successful reduction of sympathetic nervous system activity and BP in patients with DRH without affecting baroreflex control (Scheffers et al. 2010).

The Rheos pivotal Trial was a large double-blinded, randomized, placebo-controlled trial which used the same device by CVRx as above, in 265 patients with DRH (Bisognano et al. 2011). Criteria for participation included in office SBP and DBP of at least 160 mmHg and least 80 mmHg, respectively, after 1 month of a maximally tolerated antihypertensive medication treatment regimen where 3 antihypertensive were taken. Other criteria included 24-h average SBP of at least 135 mmHg and absence of clinically significant orthostatic BP. Patients were divided in a 2:1 ratio into one group that received stimulation for the 6 months following device implantation and another group that had baroreflex activation therapy initiation delayed by 6 months. At 6 months, mean SBP decreased 16 ± 29 mmHg for the patients that received baroreflex activation therapy during the first 6 months of having the device implanted, and 9 ± 29 mmHg for the delayed control group. The trial met the endpoints of sustained efficacy, baroreflex activation therapy safety, and device safety, but not acute efficacy or procedural safety (Heusser et al. 2010; Bisognano et al. 2011). After 3 years, 207 patients were still receiving follow-up, and after 5 years, 40 patients were still receiving follow-ups. Patients in these groups maintained a reduced SBP and DBP by a mean of greater than 30 mmHg and greater than 16 mmHg respectively (Heusser et al. 2010; Bisognano et al. 2011).

The Barostim Neo Trial was conducted using the Barostim neo by CVRx, the successor to the Rheos System (Hoppe et al. 2012). The device had a smaller pulse generator and only 1 lead. The smaller device permitted a smaller neck incision for placement. 30 patients had planned follow-ups at 3 and 6 months and were required to receive stable medical therapy for a minimum duration of 4 weeks with their baseline BP being established through averaging two BP readings that were separated by at least 24 h. The baroreflex activation therapy device, turned on 2 weeks post- implantation, was individually programmed to elicit optimal responses for each patient (Lohmeier et al. 2019). Patients exhibited SBP drop of 11 mmHg immediately after device implantation and 26 mmHg at the 3 month time point which was maintained into the 6 month follow-up. Limitations in the study included a requirement for office monitoring, adjustment, and procedures whenever the battery needed to be changed (Hoppe et al. 2012).

The Baroreflex Activation System Study (BRASS) was the first human proof-of-principle trial utilizing the Rheos Baroreflex Hypertension Therapy Device to combat DRH. 11 patients were enrolled in this study and had 2-year follow ups after their BAR procedures. 2 years post-BAT, the mean BP reduction exhibited by study participants was a reduction of -33/22 mmHg (Alnima et al. 2012).

The CALM-FIM study studied the efficacy of endovascular baroreflex amplification in combatting DRH with a prospective, nonrandomized trial over 3 years in 47 patients (van Kleef et al. 2022). Enrollment criteria consisted of office SBP of at least 160 mmHg and a mean 24-h ambulatory BP of at least 130/80 mmHg while being treated with at least 3 antihypertensive medications including a diuretic. 2 patients exhibited hypotension, 1 exhibited hypertension, 2 exhibited vascular access complications, 2 transient ischemic attacks took place in less than 30 days after endovascular baroreflex amplification, and 2 strokes and 1 transient ischemic attack took place 2 years after endovascular baroreflex amplification. Baseline mean office BP was 181/107 ± 17/16. 6 months post-procedure, mean office BP decreased by 25/12 mmHg, and 3 years post-procedure, mean office BP decreased by 30/12 mmHg. Baseline for mean 24-h ambulatory BP was 166/98 ± 16/15 and it exhibited a decrease of 20/11 mmHg 6 months post-procedure.

The CALM-DIEM study included 14 patients, 13 with complete data at 3-month follow-up, with DRH defined as ambulatory SBP greater than 130 mmHg while being treated with at least 3 antihypertensives including a diuretic (van Kleef et al. 2021). These patients were treated with the same endovascular baroreflex amplification used in the previous CALM studies. Eight patients had the MobiusHD installed on the right and 6 patients had it installed on the left. Office BP exhibited a significant reduction of 14/6 mmHg while mean 24-h ambulatory BP exhibited an insignificant reduction of 3/4 mmHg. The MobiusHD device by Vascular Dynamics was used in this study, and Vascular Dynamics funded the study. BP recordings were made by the Finapres NOVA by FMS (van Kleef et al. 2021).

The CALM-2 was a randomized, sham-controlled trial looking at the efficacy of endovascular baroreflex amplification on patients with DRH (van Kleef et al. 2018). Patients were randomized after meeting the following criteria: mean 24-h ambulatory SBP of 145–200 mmHg, treatment with 3–5 antihypertensives at maximally tolerated doses with the treatment regimen containing at least 1 ACE inhibitor, 1 ARB, 1 calcium channel blocker, and 1 diuretic. The trial was to last 6 months.

The CALM-START study is a randomized, sham-controlled study on patients being treated with 3–4 antihypertensives with mean 24-h ambulatory SBP measurements lying between 135–170 mmHg (Zhang et al. 2014). Patients were randomized to either MobiusHD implantation and endovascular baroreflex amplification or a sham procedure. The study looked at 3-month post procedure outcomes that were measured after a washout period. Vascular Dynamics’ MobiusHD device was used in this study. The follow-up data was not found for these ongoing clinical trials within the literature search (van Kleef et al. 2018).

### Renal denervation

Renal denervation is a minimally invasive, endovascular, investigational procedure in which a catheter is used to send ultrasound or radiofrequency waves to the arteries in the kidneys to cause ablation of the nerves in the kidneys to lower the BP by ablating afferent nerves in the kidneys project to the autonomic central nuclei which play a vital role in BP regulation (Fengler et al. 2016a; 2016b; Fengler et al. 2023; Fengler et al. 2019a; 2019b; Lurz et al. 2019; Lurz et al. 2020; Townsend et al. 2017; Denker et al. 2015). Renal denervation has been found to be safe with limited kidney injury, but induced hypotension is possible without proper monitoring during procedure (Denker et al. 2015; Denker et al. 2014).

Renal denervation has been most effective in those with high BP that has been therapy resistant (Avery et al. 2017). Those experiencing renal impairment have shown promising outcomes to renal denervation as renal impairment increases sympathetic activity (Fengler et al. 2016a; 2016b). Accessory or early bifurcated blood vessels and aortic stiffness negatively affect renal denervation outcomes. People with obesity may benefit greatly from renal denervation as increased sympathetic nervous system activity is correlated with obesity. Neither age nor gender have been shown to influence renal denervation outcomes (Fengler et al. 2016a; 2016b; Fengler et al. 2019a; 2019b).

### Renal denervation trials

The Symplicity HTN-1 study included 153 participants and was sponsored by Medtronic (Krum et al. 2014). The trial was a proof-of-concept Renal denervation study that observed BP changes from baseline after renal nerve ablation using radiofrequency was conducted using Medtronic’s Symplicity Spyral RDNcatheter (Krum et al. 2014). The patients in the trial had at least a 160-mmHg systolic BP (SBP) and were on at least three antihypertensive drugs which included a diuretic, all at ideal dosages. 111 of the 153 patients consented to follow-up for 36 months and 88 had complete data by the end of the follow up period. Significance in the data is found in the 10 mmHg drop in SBP in 69% of patients after 1 month, 81% after 6 months, 85% after 6-month, 83% after 24 months, and 93% after 36 months. The study found no significant differences at month 36 among different demographics, comorbidities, BP, and number and use of antihypertensives. The Symplicity HTN-1 trial showed promising results of renal denervation as a safe and effective treatment option for DRH (Krum et al. 2014).

The Symplicity HTN-2 study included 106 patients and was the first renal denervation study that was randomized and controlled (Esler et al. 2014). The Renal denervation procedure and the criteria for participants was the same as in the Symplicity HTN-1 study (Krum et al. 2014; Esler et al. 2014). A large portion of patients took a diuretic, a beta blocker, a calcium channel blocker, and an angiotensin-converting enzyme (ACE) inhibitor. The prior Symplicity HTN-1 study provided evidence that Renal denervation was an effective intervention to lower BP in DRH. The Symplicity HTN-2 study was intended to build upon these findings by comparing the effectiveness of Renal denervation combined with medical management on combatting DRH with the effectiveness of only medical management. The study allowed subjects in the control group to choose to receive Renal denervation after the primary endpoint evaluation that took place after the first 6 months of the study effectively creating a 36-month follow-up group and a 30-month follow-up group, and 37 patients eventually chose to do so. Overall, BP was reduced upon initial treatment with Renal denervation and maintained at 6, 12, 24, 30, and 36 months. 69 patients experienced a 34 mmHg SBP drop and a 13 mmHg diastolic BP (DBP) drop at the time of their 30 month post-procedure follow up. In the 36 month follow up group, a 33 mmHg drop in SBP and a 14 mmHg drop in DBP were observed. The catheter used in this trial was the same one used in the Symplicity HTN-1 trial. Methods used in the Symplicity HTN-2 trial confirmed the safety of Renal denervation and provided evidence of its ability to reduce BP long-term (Krum et al. 2014; Esler et al. 2014).

The conclusions from the Symplicity HTN-3 double-blinded study randomly assigned 535 patients to active Renal denervation or sham procedure in a 2:1 ratio (Bhatt et al. 2022). It included required screening with 24-h ambulatory BP monitoring in order to screen out patients with white-coat HTN. The highlight of the Symplicity HTN-3 study lies in the 6-month follow-up measurements: the active Renal denervation group exhibited a 14 mmHg drop in SBP while the sham group exhibited a 12 mmHg drop in SBP, proving active Renal denervation was not superior to treating DRH compared to the sham procedure. The 36-month follow-up showed significant improvement with a 16.5 mmHG adjusted treatment difference in ambulatory SBP between the sham and treatment group with a significant improvement of time in the therapeutic SBP range for the treatment group. There were also no significant differences in adverse effects between the groups.

The Spyral HTN OFF-MED study and the Spyral HTN ON-MED studies were international, blinded, sham-controlled trials intended to study the limitations of the Symplicity HTN-3 study and study pharmacotherapy for DRH combined with Renal denervation (Kandzari et al. 2018; Kandzari et al. 2016). These studies were conducted after the Symplicity HTN-3 trial once improved catheter technology had developed. The 100 patients in the Spyral HTN ON-MED study were on one to three antihypertensives consistently while the 120 patients in the Spyral HTN OFF-MED study participated in a three-to-four-week washout period with a three-month efficacy and safety end point without antihypertensive medications following the washout period. Both studies included randomized Renal denervation and sham-control groups. The patients that were randomized had baseline office SBP that ranged from 150 to 180 mmHg, were required to have a 24-h ambulatory SBP that was between 140 and 170 mmHg, and office DBP that was at least 90 mmHg which focused the trial on treating DRH in those with systolic-diastolic HTN and not just systolic HTN. Changes in BP were compared at 3, 6, and 12 months after Renal denervation or the sham procedure were performed on the main and branch renal arteries. In the Spyral HTN OFF-MED trial, 3 months after treatment, the patients in the sham-control group exhibited a 0.5 mmHg drop in SBP while the Renal denervation group exhibited a 5.5 mmHg drop in SBP. Also, in the Spyral HTN ON-MED trial, 6 months after treatment, the patients in the sham-control group exhibited a 1.6 mmHg drop in SBP while the Renal denervation group exhibited a 9 mmHg drop in SBP. The studies proved both the safety and the efficacy of Renal denervation as well as the therapeutic effects of pharmacotherapy. Since the improvement of the catheter technology used in Renal denervation, the Renal denervation studies can be divided into “pre-Symplicity” and “post-Symplicity” (Ram et al. 2021; Ram et al. 2014; Ram 2022; Raman et al. 2017).

The DENERHTN trial (renal denervation for Hypertension) included 106 patients with DRH that was confirmed with ambulatory BP monitoring after a 4-week treatment regimen of 1.5 mg/d of indapamide, 20 mg/d of ramipril or 300 mg/d of irbesartan, and 10 mg/d of amlodipine (Azizi et al. 2016). The trial was randomized, open-label blinded, and controlled. This trial’s 2 main purposes were 1) to analyze the therapeutic efficacy and safety of Renal denervation on ambulatory BP and 2) compare a standardized stepped antihypertensive treatment (SSAHT)—25 mg/d of spironolactone, 10 mg/d of bisoprolol. 5 mg/d of prazosin, and 1 mg/d of rilmenidine—plus renal denervation to solely a SSAHT. Patients were randomized in a 1:1 (53:53) ratio to one treatment or the other. Medications were added sequentially in the SSAHT if home BP was at least 135/85 after ramdomization. In 85 patients (40 Renal denervation, 45 control), adherence to the SSAHT was measured per the French 8-item Morisky Medication Adherence Scale (MMAS-8) via urinalysis/plasma analysis at a 6-month follow up. Patients in the Renal denervation group exhibited a mean decrease in daytime ambulatory SBP of 16.6 mmHg with a 95% confidence interval (CI) while patients in the control (SSAHT) group exhibited a mean decrease of 9 mmHg with a 95% CI. Thus, the trial concluded that Renal denervation with a SSAHT is significantly more effective at combatting DRH than a SSAHT alone. 44 of the 85 patients that had their adherence measured by the MMAS-8 were deemed nonadherent with 11 of them being completely non-adherent. Full adherence, partial nonadherence, and complete nonadherence in the Renal denervation group was exhibited by 20, 13, and 7 patients respectively while in the control group was exhibited by 21, 20, and 4 patients respectively. Thus, this trial also concluded that despite patient nonadherence of approximately 50% in both treatment groups, renal denervation plus SSAHT was more effective in combatting DRH than a SSAHT alone. Medtronic’s Symplicity flex catheter was the catheter used in this trial (Azizi et al. 2016).

The RADIANCE-HTN SOLO study was a single-blind, randomized, sham-controlled study that observed systolic-diastolic BP changes in patients with DRH after endovascular ultrasound Renal denervation (ultrasound renal denervation) using ReCor Medical’s Paradise system (Azizi et al. 2018). Patients in the study had an ambulatory BP of at least 135/85 and less than 170/105 after 4 weeks off of up to two antihypertensives. Patients to receive active Renal denervation with the Paradise system versus those to receive the sham procedure were assigned in a 1:1 ratio with the primary effectiveness endpoint being a change in daytime ambulatory SBP two months after treatment with active Renal denervation. Patients that underwent active Renal denervation with the Paradise system exhibited an 8.5 mmHg reduction in SBP while the patients that underwent the sham procedure exhibited a 2.2 mmHg reduction in SBP. The six month follow-up reported maintained the reduction in antihypertensive treatment and the reduction in ambulatory SBP compared to the sham (Azizi et al. 2019). The one year follow-up reported maintenance of medication reduction (Azizi et al. 2020). The study concluded that endovascular ultrasound renal denervation with ReCor Medical’s Paradise system was capable of lowering and maintaining a low ambulatory BP and combatting systolic-diastolic BP 2 months after treatment (Azizi et al. 2018).

The RADIANCE-HTN TRIO study was a randomized, sham-controlled study conducted to study effects of antihypertensive medication combined with ultrasound renal denervation using the ReCor Medical Paradise System used in the first 2 RADIANCE studies (Azizi et al. 2022; Azizi et al. 2021). RADIANCE_HTN TRIO studied the lasting effects of BP reduction up to 6 months rather than 2 months as in RADIANCE-II. 136 patients were included in the study. 69 underwent ultrasound renal denervation while 67 underwent the sham procedure. Patients maintained their initial antihypertensive treatment regimen until 2 months into the trial when they then were put on a standardized stepped-care antihypertensive treatment if their mean home BP was at least 135 mmHg SBP or at least 85 mmHg DBP. They remained on the standardized stepped-care antihypertensive treatment until the month 5 and it consisted of an aldosterone antagonist, a β1-blocker, a central α2-receptor blocker, and an α1-receptor blocker. 65 of the 69 ultrasound renal denervation patients exhibited a mean SBP decrease of 11.8 mmHg and 64 of the 67 sham procedure patients exhibited a mean SBP decrease of 12.3 mmHg. BP was similarly lowered in both the sham procedure group and the active ultrasound renal denervation group while the active ultrasound renal denervation group was on less medication which confirmed the efficacy once again of ReCor Medical’s Paradise System (Azizi et al. 2022; Azizi et al. 2021).

The RADIANCE-II study was a sham-controlled study that was conducted to continue research on the safety and efficacy of ultrasound renal denervation in reducing BP in hypertensive patients using the same ReCor Medical Paradise System that was used in the RADIANCE-HTN SOLO study (Azizi et al. 2023). The study had an active Renal denervation to sham-procedure ratio of 2:1 and included 224 patients that were off antihypertensives, had a BP of at least 135/85 and less than 170/105 after a 4-week washout period, suitable renal artery anatomy, and an approximated glomerular filtration rate of 40 mL/min/1.73 m (Fengler et al. 2016a; 2016b) or more. These patients were instructed to avoid antihypertensive medications until their 2-month follow-up unless excessive HTN with clinical symptoms, such as headache and dizziness, was achieved. After 2 months without antihypertensives, the 150 active ultrasound renal denervation patients exhibited a mean of a 7.9 mmHg drop in SBP while the sham-procedure population exhibited a mean SBP reduction of 1.8 mmHg which is statistically significant from the SBP drop in the active Renal denervation population.While statistical significance is important in choosing between treatment options, any significant decrease in BP can provide protection from its adverse events mentioned above, such as stroke. The RADIANCE-II study confirmed the results that were achieved from the RADIANCE-HTN SOLO study (Azizi et al. 2023).

The Oslo Renal denervation study included 19 patients ‘ results from Renal denervation or drug therapy adjustment that were randomly assigned to either Renal denervation treatment or drug therapy adjustment using Hemp Sapiens’ HOTMAN system (Bergland 2020). The study was designed to test whether Renal denervation with the Symplicity catheter is more effective than drug treatment intensification at lowering BP over the long-term (Voora et al. 2018). The Renal denervation group consisted of 9 subjects—7 males and 2 females—while the drug therapy group contained 10 males only. Patients were only eligible to participate in the Oslo Renal denervation study if ambulatory BP (ABP) remained elevated after there was a witness to the patient taking antihypertensives as prescribed. Other eligbility criteria followed the Symplicity HTN-2 study. The drug therapy group was treated with at least 3 antihypertensives with one of those being a diuretic. Calculating for renal dysfunction from drug treatment was done using the Chronic Kidney Disease Epidemiology Collaboration for measuring creatinine levels. Patients were to have 1-month, 3-month, 6-month, 1-year. 3-year, and 7-year follow ups to study Renal denervation effects vs drug therapy effects over the long-term. Patients on drug therapy who were still in HTN at their first year follow up had their treatment regimen modified. At the time of the 6-month follow-up, patients’ BPs were not lowered significantly in either group, but by the 7-year follow-up, mean systolic ABP had increased in the Renal denervation group from 142 ± 10 to 145 ± 15 mmHg and, in the drug adjustment group, from 133 ± 11 to 137 ± 13 mmHg. The study concluded that Renal denervation is equally as effective as but not more effective than drug treatment at combatting DRH 7 years after the Renal denervation procedure (Undrum Bergland et al. 2021).

The INSPiRED (Investigator-Steered Project on Intravascular Denervation for Management of Treatment-Resistant Hypertension) study included 15 patients that met the qualifications to participate in the study which were having BP of at least 130/80 mmHg despite being treated with at least 3 antihypertensive medications (Jacobs et al. 2017). The study aimed to analyze their 6-month follow up results to observe if either Renal denervation using the EnligHTN system along with regular hypertensive treatment or purely regular hypertensive treatment was a superior treatment to the other. Patients were randomized to treatment to either Renal denervation using the EnligHTN system along with regular antihypertensive treatment or purely regular antihypertensive treatment in a 1:1 ratio. Primary efficacy and safety endpoints in this study were 19.5/10.4 mmHg and 2.5 mL/min/1.73 m (Fengler et al. 2016a; 2016b) respectively. Office BP was measured using Omron Health Care’s Omron HEM-907 system. Renal denervation along with hypertensive treatment was superior to hypertensive treatment alone in significantly decreasing nighttime BP but not office or daytime ABP (Jacobs et al. 2017; Kjeldsen et al. 2014).

PRAGUE-15 was a randomized and multicenter 2-year study included data on how Renal denervation compared with intensified pharmacological treatment (PHAR) specifically using spironolactone in 86 patients (42:44) (Rosa et al. 2015; Rosa et al. 2016; Rosa et al. 2017). True resistant hypertension was defined as SBP of at least 140 mmHg, ambulatory 24-h mean SBP of at least 130 mmHg, having been treated with at least 3 antihypertensive medications including a diuretic, no secondary hypertension, and compliance with an antihypertensive treatment regimen. Baseline SBP was 159 ± 17 for the 42 Renal denervation patients and 155 ± 17 mmHg for the 44 spironolactone patients. At 2-years, 86 patients showed an insignificant difference between Renal denervation and PHAR with a 9.1 mmHg BP reduction in the Renal denervation population and a 10.9 mmHg SBP reduction in the PHAR population. Nonetheless, the study concluded that PHAR with spironolactone is more effective at combatting DRH than Renal denervation due to the larger decrease in SBP. The Renal denervation catheter used in this study was Medtronic’s Symplicity renal denervation System (Rosa et al. 2015; Rosa et al. 2016; Rosa et al. 2017).

RADIOSOUND-HTN was a three-arm study looking at 3 different styles of Renal denervation procedures and their effectiveness at combatting DRH at 3 months post-procedure: radiofrequency Renal denervation (RF-Renal denervation) of the main renal arteries (RFM-Renal denervation), RF-Renal denervation of the main renal arteries but also the side branches and accessories (RFB-Renal denervation), and endovascular ultrasound renal denervation of the main renal artery (USM-Renal denervation) (Fengler et al. 2023; Fengler et al. 2019a; 2019b). The study was single-blinded and randomized. This study enrolled 120 patients with 39 of them receiving RFM-Renal denervation, 39 receiving RFB-Renal denervation, and 42 receiving USM-Renal denervation, but 1 patient in the RFM-Renal denervation and 2 patients in the RFB-Renal denervation were not followed up with, so final 3-month data was available in 117 patients. Patient eligibility requirements consisted of daytime SBP of at least 135 mmHg during ambulatory blood pressure monitoring (ABPM) despite treatment with 3 antihypertensive medications at a minimum of 50% of the max dose for HTN treatment with one of them being a diuretic. A significant BP reduction was considered at least a 5-mmHg reduction in daytime SBP on ABPM at 3 months. Daytime BP was reduced in all patients by 9.5/6.3 ± 12.3/7.8. No significant difference in daytime SBP reduction was present between the 2 RF-Renal denervation groups, but a significant difference was seen between ultrasound renal denervation and RF-Renal denervation of the main renal artery as the ultrasound renal denervation group exhibited a reduction of 13.2 ± 13.7 mmHg and the RF-Renal denervation group exhibited a reduction of 6.5 ± 10.3 mmHg. The reduction in daytime SBP in the RFB-Renal denervation group was one of 8.3 ± 11.7. The study concluded that ultrasound renal denervation is more effective at combatting DRH than RF-Renal denervation. The 6 and 12 month follow up data confirmed that ultrasound renal denervation produces a superior reduction in systolic BP over radiofrequency ablation (Fengler et al. 2023). The RF-Renal denervation catheter used in this study was the multipolar Symplicity Spyral catheter by Medtronic and the ultrasound renal denervation catheter used was the Paradise catheter by ReCor (Fengler et al. 2023; Fengler et al. 2019a; 2019b).

TARGET BP OFF-MED and TARGET BP I were randomized, blinded, and sham-controlled studies that attempted to combat DRH using alcohol-mediated Renal denervation in the presence and absence of antihypertensives (Pathak et al. 2023). 106 patients were enrolled that had a 24-h SBP of 135–170 mmHg, and office SBP of 140–180 mmHg, and DBP of at least 90 mmHg while on 0–2 antihypertensive medications. The study lasted 8 weeks with the baseline post-washout BP measurements being 159.4/100.4 ± 10.9/7 mmHg in the Renal denervation group and 160.1/98.3 ± 11.0/6.1 mmHg in the sham group. Mean 24-h SBP change at 8 weeks was -2.9 ± 7.4 mmHg in the Renal denervation group and -1.4 ± 8.6 mmHg. The groups had no safety differences. At a blinded 12-month follow up after medication escalation, the groups maintained similar office SBP: the Renal denervation group had a mean office SBP of 147.9 ± 18.5 mmHg while the sham group had a mean office BP of 147.8 ± 15.1 mmHg with the Renal denervation group taking less medications. While alcohol mediated Renal denervation was proven safe by these studies, it did not produce significant differences between the Renal denervation and the sham group. The Peregrine Catheter by Ablative Solutions was used for these studies (Pathak et al. 2023).

The REQUIRE trial looked at Renal denervation efficacy versus sham efficacy at 3 months in Japanese and South Korean patients with DRH as the Japanese and South Korean population possess a phenotype different from the Caucasian population associated with hypertension (Kario et al. 2022). Cardiovascular risk differs among different races, too. This study enrolled 143 patients who were randomized to either Renal denervation or sham procedure (72:71) who had hypertension resistant to at least 3 antihypertensives, including a diuretic, at maximally tolerated doses. Patients also had an average seated office BP of at least 150/90 mmHg. While the procedures were therapeutic, there was no significant difference between the 242-h ambulatory BP reduction in the Renal denervation group and the sham group as the Renal denervation group exhibited a reduction of 6.6 mmHg reduction and the sham group exhibited a 6.5 mmHg reduction. Medication load did not differ between the groups. The catheter used in this study was ReCor Medical’s Paradise Renal denervation system (Kario et al. 2022).

The Symplicity HTN-Japan trial was a randomized, controlled trial that was the first to study Renal denervation in a Japanese patient population (Kario et al. 2019). The trial included 22 patients receiving Renal denervation with 19 being a control group and 11 crossing over from medical treatment to Renal denervation at 6 months after randomization. DRH in this study was defined as SBP of at least 160 mmHg and 24-h ambulatory SBP of at least 135 mmHg while on at least 3 antihypertensive medications at maximally tolerated doses for 6 weeks before being enrolled in the study. At 36 months, the Renal denervation group exhibited a reduction in office SBP of 32.8 ± 20.1 mmHg and a reduction in office DBP of 15.8 ± 12.6. At 30 months, the Renal denervation crossover group exhibited an SBP reduction of 26.7 ± 18.9 and a DBP reduction of 12.7 ± 11.8 mmHg. The trial concluded that Renal denervation exhibits sustained efficacy in reducing BP up to 36 months post-procedure. The Symplicity flex catheter by Medtronic was used in this trial to conduct renal denervation (Kario et al. 2019).

The WAVE IV study was a nonrandomized, sham-controlled, double-blinded study that was conducted to verify the efficacy of external bilateral ultrasound renal denervation in treating DRH by comparing it to the sham procedure (Schmieder et al. 2018). 81 eligible patients were enrolled in the study with the criteria of having an office BP of at least 160 mmHg and a 24-h ambulatory BP of at least 135 mmHg while being treated with at least 3 antihypertensive medications.12 weeks post-procedure, the uRDN group exhibited a 13.2 ± 20 mmHg office SBP reduction while the sham group exhibited an 18.9 ± 14 mmHg office SBP reduction. The difference between the groups’ office SBP reductions was significant, but the difference between the groups’ office DBP reductions was not. At the same time point, the uRDN group exhibited a 4.95 ± 12 mmHg office DBP reduction while the sham group exhibited a 6.5 ± 11 mmHg office DBP reduction. 24 weeks post-procedure, the uRDN group exhibited a 12.8 ± 16 mmHg office SBP reduction while the sham group exhibited a 23 ± 20 mmHg office SBP reduction. 24 weeks post-procedure, the uRDN group exhibited a 5.1 ± 15 mmHg office DBP reduction while the sham group exhibited an 8.9 ± 12 mmHg office DBP reduction. At the same time point, 24-h ambulatory BP changes were measured. The uRDN group exhibited a 7.11 ± 13 mmHg 24-h ambulatory SBP reduction while the sham group exhibited a 5.90 ± 15 mmHg 24-h ambulatory SBP reduction. At the same time point, the uRDN group exhibited a 5.0 ± 9.9 mmHg 24-h ambulatory DBP reduction while the sham group exhibited a 4.5 ± 9.5 mmHg 24-h ambulatory DBP reduction. The study concluded that the efficacy of external bilateral uRDN was not significantly greater than the sham procedure at combatting DRH. The Surround Sound System by KonaMedical was used in this study (Schmieder et al. 2018).

The ISAR-denerve study observed the efficacy of RDN in patients that have had a renal transplant (Schneider et al. 2015). Approximately 70–90% of patients exhibit arterial hypertension or need treatment with antihypertensives after renal transplantation, so this study aimed to observe the effects of RDN on the native kidneys of renal transplantation patients in hope of reducing BP and sympathetic overactivity. 18 patients were randomized (1:1) to RDN or medical treatment without RDN and had their office SBP recorded after 6 months. These patients had to have a diagnosis of persistent hypertension 6 months after their renal transplantation, an office SBP of at least 140 mmHg while being treated with at least 3 antihypertensives including a diuretic and have no medication changes for at least 2 weeks prior to being enrolled to be eligible for trial participation. The RDN group exhibited a reduction in office SBP of 23.3 ± 14.5 mmHg and a reduction in nocturnal BP of -10.38 ± 12.8 mmHg, but no BP changes were measured in the daytime. The trial concluded that RDN is effective in combatting DRH in renal transplantation patients. The study utilized Medtronic’s Symplicity Flex cather (Schneider et al. 2015).

The DENERVHTA study was a randomized controlled study that compared the efficacy of RDN versus the efficacy of 50 mg of spironolactone at combatting DRH (Oliveras et al. 2016). The study produced complete 6-month data from 24 patients with an office SBP of at least 150 mmHg and a 24-h SBP of at least 140 mmHg while being treated with at least 3 antihypertensives, including one diuretic and no aldosterone antagonists, at maximally tolerated doses. 11 patients were randomized to RDN and 13 were randomized to spironolactone treatment. The study lasted 6 months. At 6 months, the RDN group exhibited a reduction in 24-h SBP and 24-h DBP of 5.7 mmHg and 3.7 mmHg, respectively. The spironolactone group exhibited a reduction in 24-h SBP and 24-h DBP of 23.6 mmHg and 10.2 mmHg, respectively. The spironolactone group saw a greater reduction in 24-h SBP and 24-h DBP than the RDN group concluding that spironolactone was more effective at combatting DRH than RDN (Oliveras et al. 2016).

A study conducted in Lithuania aimed to analyze long-term effects of RDN on BP by observing changes at 48 months post-procedure and aimed to analyze the impact of the number of antihypertensive medications taken on BP changes (Juknevicius 2021). Data was obtained from 49 patients in the final analysis. Inclusion criteria included renal arteries greater than 3 mm in diameter, greater than 20 mm in length, no significant atherosclerosis, no arterial abnormalities, no stenosis or history of renal artery stenting, and greater than 18 years old. Exclusion criteria included history of acute myocardial infarction, irregular angina, history of a cerebrovascular accident within the previous 6 months, hemodynamically significant valvular disease, chronic kidney disease, and secondary hypertension. The ablation process in this study was different than in the studies described so far. The RDN catheter was placed on a wire through the guiding catheter to keep the electrode portion of the catheter straight while being inserted. Once the electrode portion of the catheter was in place, the wire was withdrawn, and the electrode portion of the catheter coiled up and stuck to the inner arterial walls to which it coiled up to. Radiofrequency ablation was performed once the electrodes were stuck in place. RDN then proceeded following regular RDN protocol. Aspirin or clopidogrel were given for a minimum of one-month post-procedure to prevent aggregation. Follow-ups were conducted at 3, 6, 12, and 48 months post-procedure, and office and 24-h ambulatory BP measurements were taken. Study limitations include the smaller sample size. The median office BP among the patients before RDN was 180/110 mmHg and patients were on an average of 6.25 antihypertensive medications. At 3, 6, 12, 24, and 48 months post-procedure, median office BP measurements were 162.5/94.5, 151/89.5, 153/93.5, 169/95, and 165/95, respectively. The authors found a positive correlation between number of pills taken and BP, but no significant differences in 24-h BP among different groups of participants—patients that took 1–5 pills, 6–10 pills, or more than 10 pills. The Symplicity FlexTM cather and the Symplicity G2TM generator or the Smyplicity SpyralTM catheter and the Symplicity G3TM generator by Medtronic were used in this study. Limitations in this study included its sample size, lack of a control group, no sympathetic nervous activity data, and lack of plasma or urine drug concentration data from the study participants.

### Sympathectomy

Surgical lumbar sympathectomy is performed via retroperitoneal access to the sympathetic trunk via a 12 to 15 mm incision across the internal and external obliques (Beglaibter et al. 2002). Once access to the sympathetic chain is achieved, clips were placed on the L2 and L4 sympathetic nerves which were then transected to sever and remove the sympathetic chain (Beglaibter et al. 2002). This removal reduces peripheral resistance therefore increasing the volume of anastomotic collateral arteries consequently reducing BP (van der Stricht 1979). The procedure often leaves patients with postural hypotension, syncope, and impotence, therefore, Renal denervation is the more favorable procedure today (Obi et al. 2023). Lumbar sympathectomies induce permanent autonomic nervous system balance towards parasympathetic tone and vasodilation due to the lack of adrenergic capabilities. This vasodilation reduces BP but can result in chronic postural hypotension if prolonged (van Lieshout et al. 1990).

The sympathectomy procedure has three possible approaches: transperitoneal anterior, extraperitoneal anterior, and posterior extraperitoneal (Collin et al. 1994). The procedure can also be performed using open, laparoscopic, or percutaneous approaches. The procedure consists of a lumbar sympathetic nerve block that results in permanent vasodilation without affecting output to muscles that may be in use (Karanth 2016). Sympathetic ganglia at the lumbar level that play roles in vasoconstriction are destroyed to increase arterial perfusion, thus widening arteries and reducing BP (Karanth 2016). Lumbar sympathectomies can be chemical percutaneous, laparoscopic, or surgical using an open approach. The numerous irreversible adverse events described above led to the retirement of sympathectomies in the treatment of DRH. There are clinical trials of sympathectomies available in the literature.

### DRG stimulation

DRG stimulation has been shown to reduce sympathetic nerve output and BP (Sverrisdottir et al. 2020). Proper stimulation is achieved through continuous electrical stimulation with an electrode that is connected to a subcutaneous pulse generator implanted via an epidural approach. Stimulation of the DRG at L1 can influence BP as the sympathetic efferent nerves project from the lumbar sympathetic chain to the adrenal glands (Sverrisdottir et al. 2020). These sympathetic efferent nerves assist in BP control via non-adrenergic mechanisms including dopamine, neuropeptide, and purine modulation (Mathias 1991). There is evidence for left sided DRG stimulation lowering BP after six months from a hypertensive baseline and has proven to remain effective two years post-installation of the DRG stimulator, while right side DRG stimulation has not. The physiology behind this is currently unknown. No significant difference was identifiable between BP measurements during the 6-month follow-up versus the 2-year follow-up, indicating DRG stimulation may decrease BP post-operatively but not throughout the following years. One study including data from 14 patients (7 males, 7 females), with a minimum of 2 years of refractory neuropathy despite treatment with 3 analgesics of different classes, observed acute decreases of BP with DRG stimulation on versus off. The DRG Axium lead by Spinal Modulation, now made by Abbot inc., was used in this study. Current contraindications DRG stimulation include neuroforaminal stenosis and anatomical lack of access (Sverrisdottir et al. 2020).

### Spinal cord stimulation

Spinal cord stimulation is currently only indicated for chronic pain, but pilot ancillary studies have shown that spinal cord stimulation may reduce BP in hypertensive patients by decreasing postganglionic muscle sympathetic nerve activity which is elevated in hypertensive patients (Memar et al. 2023a; 2023b; Holwerda et al. 2021; Holwerda et al. 2018). spinal cord stimulation systems consist of a pulse generator with 1 or 2 leads containing up to 8 contacts each. They can be controlled remotely by the patient through a wireless handheld device. Low-frequency spinal cord stimulation has been shown to reduce BP in patients with DRH, but high-frequency spinal cord has limited evidence to show its efficacy in BP reduction (Memar et al. 2023a; 2023b).

Schultz et al. aimed to investigate the effect of acute dorsal spinal cord stimulation on mean arterial pressure and heart rate. (Schultz 2007) Data from 8 normotensive patients were included in the final analysis of this study. A cold pressor test was used to induce a hypertensive state. A Vasotrac device was used to measure BP and heart rate at the wrist. The quadripolar leads used in this study resided in the epidural spaces at T1-T2 or T5-T6. Stimulation location was random, and stimulation was conducted before and during the cold pressor test. During the cold pressor test, a patient’s hand was submerged in ice water for 2 min. The cold pressor test with no stimulation elevated mean arterial pressure by 9.4 ± 3.8 mmHg. Spinal cord stimulation with no cold pressor test elevated mean arterial pressure by 9.2 ± 5 mmHg at T1-T2 and by 10.7 ± 8.4 mmHg at T5-T6. During stimulation at T5-T6, the cold pressor test significantly increased mean arterial pressure by 5.9 ± 7.1 mmHg compared to the 9.4 ± 3.8 mmHg increase induced by the cold pressor test alone. The study concluded that spinal cord stimulation at T1-T2 or T5-T6 did not alter mean arterial pressure to a significant degree, but it did increase mean arterial pressure during the cold pressor test when stimulating at T5-T6. This finding is inconsistent with existent literature but found that the procedure itself is safe.

Schultz. et al. later aimed to investigate the acute effects of spinal cord stimulation on mean arterial pressure at two different spinal cord stimulation strengths, 100% and 80%, during cold pressor tests. (Schultz et al. 2011) Three cold pressor tests were conducted: one without stimulation, one with 80% stimulation, and one with 100% stimulation. 6 hypertensive and 9 normotensive patients were included in this study. Mean arterial pressure was measured during a cold pressor test via photoplethysmography at the finger using a Finometer called Model 1 by Finapress Medical Systems. Stimulation was conducted at T5-T6 using an external programmer and leads implanted under sedation 3 days before the study took place. Patients’ right hands were submerged in ice water for 90 s with mean arterial pressure data being acquired for 30 s prior to submersion to acquire a baseline. The normotensive group’s mean arterial pressure was elevated 16 mmHg during the placebo phase, 18 mmHg during 80% stimulation, and 10.5 mmHg during 100% stimulation. The hypertensive group’s mean arterial pressure was elevated 26.8 mmHg during the placebo phase, 20 mmHg during 80% stimulation, and 17 mmHg during 100% stimulation. While change in mean arterial pressure occurred, no changes were significant. This study concluded that spinal cord stimulation did not elicit any significant changes in mean arterial pressure and that more insight into effects of chronic spinal cord stimulation is needed.

In one retrospective study, 132 patients’ electronic medical records from The University of Kansas Health System confirmed that low frequency-spinal cord stimulation lowers BP while studying the effects of spinal cord stimulation on chronic pain treatment (Memar et al. 2023a; 2023b). BP measurements from 3 months prior to and 3 months post spinal cord stimulation implantation were averaged for analysis. 62 patients had received low frequency-spinal cord stimulation and 70 had received high frequency-spinal cord stimulation. Stimulators were surgically implanted from T7-T10 along the posterior dorsal column. A clinically significant 8 mmHg reduction in SBP was exhibited in patients upon implantation of the stimulators. Patients were separated into 3 groups: normal/elevated BP (BP less than 130/80), HTN-1 (BP more than 130/80), and HTN-2 (BP more than 140/90). Following high frequency-spinal cord stimulation, the normal/elevated and HTN-1 group exhibited a SBP increase of 3 ± 8 mmHg, but the HTN-2 group exhibited an SBP decrease of 7 ± 8 mmHg. DBP did not show significant changes with high frequency-spinal cord stimulation. Following low frequency-spinal cord stimulation, the normal/elevated group exhibited a SBP increase of 5 ± 13 mmHg while the HTN-2 group exhibited a drop of 8 ± 14 mmHg. DBP did not show significant changes with low frequency-spinal cord stimulation. High frequency-spinal cord stimulation produced more consistent results and therefore may produce effects that are more predictable than low frequency-spinal cord stimulation. Patients with higher baseline BP responded better than those with lower BP to spinal cord stimulation treatment. This study concluded that both low frequency- and high frequency-spinal cord stimulation have proven to be effective at combatting DRH.

### Considerations for future clinical trials for device therapies towards autonomic nervous system and blood pressure modulation

The effectiveness of renal denervation has been demonstrated through multiple studies, thus further investigation into the intricacies of the treatment should not be the main direction of future study. Other approaches are essential for patients with contraindications to renal denervation such as early bifurcation of renal vessels and aortic stiffening. The novelty of the DRG stimulation and spinal cord stimulation trial shows promise as an avenue for further prospective clinical and mechanistic exploration in the realm of DRH treatment. There are recent studies of spinal cord stimulation in spinal cord injury patients to treat hypotension (Squair et al. 2021). We hope that further study of spinal cord stimulation will enable treatment of other hemodynamic instabilities, in this case DRH. While renal denervation and carotid body stimulation show the highest efficacy in lowering blood pressure and have both been analyzed by multiple randomized control trials and meta-analyses to confirm the statistical significance of the studies, they both have contraindications leading to the necessity of other treatment modalities. This review suggests that further exploration of double blinded, randomized control trials of spinal modulation, including both spinal cord stimulation and DRG stimulation, would add necessary literature needed to provide meta-analyses. To provide the most statistically significant insight into the efficacy and safety of DRG stimulation and spinal cord stimulation as treatment for DRH, the randomized control trials should be multi-center international registries to battle against DRH.

## Limitations

Publication bias and selection bias are inherent to the nature of systematic reviews due to probability that published data is more likely to provide statistically significant results. This bias could affect the inclusion of data suggesting poor outcomes, since this data is less likely to be reported. Some articles that may fit our inclusion/exclusion criteria may not be retrieved from the literature search. This should be diminished through adherence PRISMA guidelines. Another limitation to this review is the small number of spinal cord stimulation and DRG trials, preventing rigorous statistical analysis of the outcomes. The high number of renal denervation and carotid body stimulation trials provides the most reliable data with sufficient evidence on their efficacy. More trials provide a greater ability to perform further statistical analyses on this data, further enhancing the efficacy of the data. The paucity of data in spinal stimulation, both DRG and spinal cord stimulation, prevents further analysis and comparison of the intervention to the other battle-hardened treatment modalities. This further illustrates the necessity for future trials to primarily include spinal stimulation with standardized reporting to provide data for statistically significant comparison of treatment modality of DRH.

## Conclusion

This review summarizes the outcome of the clinical trials that treated DRH using neuromodulatory techniques, including carotid body stimulation, renal denervation, and sympathectomies while discussing the importance of emerging modulator therapies including DRG stimulation and spinal cord stimulation. The treatment of DRH using neuromodulatory techniques has shown significantly effective results in both renal denervation and carotid body stimulation. These techniques are also clinically significant by lowering the risk for adverse events caused by high blood pressure. However, many patients who are contraindicated for these procedures due to renal artery stenosis and other diseases need other avenues of therapy. Future studies should focus on discerning the efficacy of spinal cord and DRG stimulation approaches for this patient population. Future multi-center prospective registries, as well as mechanistic studies in basic science, are required to gain insight of spinal cord stimulation as treatment due to the paucity of significant data available.

## Data Availability

No datasets were generated or analysed during the current study.

## Abbreviations

BP: Blood Pressue
DRH: Drug-Resistant Hypertension

## References

[CR1] Alnima T, de Leeuw PW, Kroon AA. Baroreflex activation therapy for the treatment of drug-resistant hypertension: new developments. Cardiol Res Pract. 2012;2012:587194. 10.1155/2012/587194. 22762007 10.1155/2012/587194 PMC3384947

[CR2] Avery MC, Krichmar JL. Neuromodulatory systems and their interactions: a review of models, theories, and experiments. Front Neural Circuits. 2017;11:108. 10.3389/fncir.2017.00108. 29311844 10.3389/fncir.2017.00108 PMC5744617

[CR3] Azizi M, et al. Adherence to antihypertensive treatment and the blood pressure-lowering effects of renal denervation in the renal denervation for hypertension (DENERHTN) trial. Circulation. 2016;134:847–57. 10.1161/CIRCULATIONAHA.116.022922. 27576780 10.1161/CIRCULATIONAHA.116.022922

[CR4] Azizi M, et al. Endovascular ultrasound renal denervation to treat hypertension (RADIANCE-HTN SOLO): a multicentre, international, single-blind, randomised, sham-controlled trial. Lancet. 2018;391:2335–45. 10.1016/S0140-6736(18)31082-1. 29803590 10.1016/S0140-6736(18)31082-1

[CR5] Azizi M, et al. Six-month results of treatment-blinded medication titration for hypertension control after randomization to endovascular ultrasound renal denervation or a sham procedure in the RADIANCE-HTN SOLO trial. Circulation. 2019;139:2542–53. 10.1161/CIRCULATIONAHA.119.040451. 30880441 10.1161/CIRCULATIONAHA.119.040451

[CR6] Azizi M, et al. 12-Month results from the unblinded phase of the RADIANCE-HTN SOLO trial of ultrasound renal denervation. JACC Cardiovasc Interv. 2020;13:2922–33. 10.1016/j.jcin.2020.09.054. 33357531 10.1016/j.jcin.2020.09.054

[CR7] Azizi M, et al. Ultrasound renal denervation for hypertension resistant to a triple medication pill (RADIANCE-HTN TRIO): a randomised, multicentre, single-blind, sham-controlled trial. Lancet. 2021;397:2476–86. 10.1016/S0140-6736(21)00788-1. 34010611 10.1016/S0140-6736(21)00788-1

[CR8] Azizi M, et al. Effects of renal denervation vs sham in resistant hypertension after medication escalation: prespecified analysis at 6 months of the RADIANCE-HTN TRIO randomized clinical trial. JAMA Cardiol. 2022;7:1244–52. 10.1001/jamacardio.2022.3904. 36350593 10.1001/jamacardio.2022.3904 PMC9647563

[CR9] Azizi M, et al. Endovascular ultrasound renal denervation to treat hypertension: the RADIANCE II randomized clinical trial. JAMA. 2023;329:651–61. 10.1001/jama.2023.0713. 36853250 10.1001/jama.2023.0713 PMC9975904

[CR10] Beglaibter N, Berlatzky Y, Zamir O, Spira RM, Freund HR. Retroperitoneoscopic lumbar sympathectomy. J Vasc Surg. 2002;35:815–7. 10.1067/mva.2002.121130. 11932687 10.1067/mva.2002.121130

[CR11] Bergland OU, et al. The randomised Oslo study of renal denervation vs. Antihypertensive drug adjustments: efficacy and safety through 7 years of follow-up. Blood Press. 2021;30:41–50. 10.1080/08037051.2020.1828818. 33030064 10.1080/08037051.2020.1828818

[CR12] Bhatt DL, et al. Long-term outcomes after catheter-based renal artery denervation for resistant hypertension: final follow-up of the randomised SYMPLICITY HTN-3 Trial. Lancet. 2022;400:1405–16. 10.1016/S0140-6736(22)01787-1. 36130612 10.1016/S0140-6736(22)01787-1

[CR13] Bisognano JD, et al. Baroreflex activation therapy lowers blood pressure in patients with resistant hypertension: results from the double-blind, randomized, placebo-controlled rheos pivotal trial. J Am Coll Cardiol. 2011;58:765–73. 10.1016/j.jacc.2011.06.008. 21816315 10.1016/j.jacc.2011.06.008

[CR14] Charkoudian N, Rabbitts JA. Sympathetic neural mechanisms in human cardiovascular health and disease. Mayo Clin Proc. 2009;84:822–30. 10.4065/84.9.822. 19720780 10.4065/84.9.822 PMC2735432

[CR15] Collin J, Gordon A. Lumbar sympathectomy. Lancet. 1994;344:1507. 7968141

[CR16] Converse RL Jr, et al. Sympathetic overactivity in patients with chronic renal failure. N Engl J Med. 1992;327:1912–8. 10.1056/NEJM199212313272704. 1454086 10.1056/NEJM199212313272704

[CR17] Denker MG, Cohen DL. Use of continuous positive airway pressure for sleep apnea in the treatment of hypertension. Curr Opin Nephrol Hypertens. 2014;23:462–7. 10.1097/MNH.0000000000000047. 24992567 10.1097/MNH.0000000000000047 PMC4227307

[CR18] Denker MG, Cohen DL. Resistant hypertension and renal nerve denervation. Methodist Debakey Cardiovasc J. 2015;11:240–4. 10.14797/mdcj-11-4-240. 27057294 10.14797/mdcj-11-4-240 PMC4814011

[CR19] Doroszko A, Janus A, Szahidewicz-Krupska E, Mazur G, Derkacz A. Resistant hypertension. Adv Clin Exp Med. 2016;25:173–83. 10.17219/acem/58998. 26935512 10.17219/acem/58998

[CR20] Esler MD, et al. Catheter-based renal denervation for treatment of patients with treatment-resistant hypertension: 36 month results from the SYMPLICITY HTN-2 randomized clinical trial. Eur Heart J. 2014;35:1752–9. 10.1093/eurheartj/ehu209. 24898552 10.1093/eurheartj/ehu209 PMC5994826

[CR21] Fengler K, Rommel KP, Okon T, Schuler G, Lurz P. Renal sympathetic denervation in therapy resistant hypertension - pathophysiological aspects and predictors for treatment success. World J Cardiol. 2016a;8:436–46. 10.4330/wjc.v8.i8.436. 27621771 10.4330/wjc.v8.i8.436 PMC4997524

[CR22] Fengler K, et al. Renal denervation improves exercise blood pressure: insights from a randomized, sham-controlled trial. Clin Res Cardiol. 2016b;105:592–600. 10.1007/s00392-015-0955-8. 26728060 10.1007/s00392-015-0955-8

[CR23] Fengler K, et al. Renal denervation in isolated systolic hypertension using different catheter techniques and technologies. Hypertension. 2019a;74:341–8. 10.1161/HYPERTENSIONAHA.119.13019. 31203726 10.1161/HYPERTENSIONAHA.119.13019

[CR24] Fengler K, et al. A three-arm randomized trial of different renal denervation devices and techniques in patients with resistant hypertension (RADIOSOUND-HTN). Circulation. 2019b;139:590–600. 10.1161/CIRCULATIONAHA.118.037654. 30586691 10.1161/CIRCULATIONAHA.118.037654

[CR25] Fengler K, et al. 6- and 12-month follow-up from a randomized clinical trial of ultrasound vs radiofrequency renal denervation (RADIOSOUND-HTN). JACC Cardiovasc Interv. 2023;16:367–9. 10.1016/j.jcin.2022.10.058. 36792266 10.1016/j.jcin.2022.10.058

[CR26] Gronda E, et al. Baroreflex activation therapy: a new approach to the management of advanced heart failure with reduced ejection fraction. J Cardiovasc Med (Hagerstown). 2017;18:641–9. 10.2459/JCM.0000000000000544. 28737621 10.2459/JCM.0000000000000544 PMC5555968

[CR27] Hamdidouche I, et al. Clinic versus ambulatory blood pressure in resistant hypertension: impact of antihypertensive medication nonadherence: a post hoc analysis the DENERHTN study. Hypertension. 2019;74:1096–103. 10.1161/HYPERTENSIONAHA.119.13520. 31995406 10.1161/HYPERTENSIONAHA.119.13520

[CR28] Hausberg M, Grassi G. Mechanisms of sympathetic overactivity in patients with chronic renal failure: a role for chemoreflex activation? J Hypertens. 2007;25:47–9. 10.1097/HJH.0b013e3280119286. 17143171 10.1097/HJH.0b013e3280119286

[CR29] Herlitz H, Hjemdahl P, Volkmann R, Jensen G, Aurell M. Renal and systemic sympathetic counterregulation in response to vasodilators in renovascular hypertension. Clin Sci (Lond). 1993;84:41–5. 10.1042/cs0840041. 8382132 10.1042/cs0840041

[CR30] Heusser K, et al. Carotid baroreceptor stimulation, sympathetic activity, baroreflex function, and blood pressure in hypertensive patients. Hypertension. 2010;55:619–26. 10.1161/HYPERTENSIONAHA.109.140665. 20101001 10.1161/HYPERTENSIONAHA.109.140665

[CR31] Holwerda SW, Holland MT, Reddy CG, Pierce GL. Femoral vascular conductance and peroneal muscle sympathetic nerve activity responses to acute epidural spinal cord stimulation in humans. Exp Physiol. 2018;103:905–15. 10.1113/EP086945. 29603444 10.1113/EP086945 PMC5984152

[CR32] Holwerda SW, Holland MT, Green AL, Pearson ACS, Pierce GL. Dissociation between reduced pain and arterial blood pressure following epidural spinal cord stimulation in patients with chronic pain: A retrospective study. Clin Auton Res. 2021;31:303–16. 10.1007/s10286-020-00690-5. 32323062 10.1007/s10286-020-00690-5 PMC8456508

[CR33] Hoppe UC, et al. Minimally invasive system for baroreflex activation therapy chronically lowers blood pressure with pacemaker-like safety profile: results from the Barostim neo trial. J Am Soc Hypertens. 2012;6:270–6. 10.1016/j.jash.2012.04.004. 22694986 10.1016/j.jash.2012.04.004

[CR34] Hosseini-Dastgerdi H, Kharazmi F, Pourshanazari AA, Nematbakhsh M. Renal denervation influences angiotensin II types 1 and 2 receptors. Int J Nephrol. 2022;2022:8731357. 10.1155/2022/8731357. 36262553 10.1155/2022/8731357 PMC9576444

[CR35] Jacobs L, et al. Results of a randomized controlled pilot trial of intravascular renal denervation for management of treatment-resistant hypertension. Blood Press. 2017;26:321–31. 10.1080/08037051.2017.1320939. 28489464 10.1080/08037051.2017.1320939

[CR36] Johnson MD, et al. Neuromodulation for brain disorders: challenges and opportunities. IEEE Trans Biomed Eng. 2013;60:610–24. 10.1109/TBME.2013.2244890. 23380851 10.1109/TBME.2013.2244890 PMC3724171

[CR37] Jordan J, et al. Contrasting effects of vasodilators on blood pressure and sodium balance in the hypertension of autonomic failure. J Am Soc Nephrol. 1999;10:35–42. 10.1681/ASN.V10135. 9890307 10.1681/ASN.V10135

[CR38] Juknevicius V, et al. Long-Term Effects of Renal Artery Denervation. Medicina (Kaunas). 2021;57:662. 10.3390/medicina57070662. 34199107 10.3390/medicina57070662 PMC8305318

[CR39] Karanth VK, Karanth TK, Karanth L. Lumbar sympathectomy techniques for critical lower limb ischaemia due to non-reconstructable peripheral arterial disease. Cochrane Database Syst Rev. 2016;12:CD011519. 10.1002/14651858.CD011519.pub2. 27959471 10.1002/14651858.CD011519.pub2 PMC6463847

[CR40] Kandzari DE, et al. The SPYRAL HTN global clinical trial program: rationale and design for studies of renal denervation in the absence (SPYRAL HTN OFF-MED) and presence (SPYRAL HTN ON-MED) of antihypertensive medications. Am Heart J. 2016;171:82–91. 10.1016/j.ahj.2015.08.021. 26699604 10.1016/j.ahj.2015.08.021

[CR41] Kandzari DE, et al. Effect of renal denervation on blood pressure in the presence of antihypertensive drugs: 6-month efficacy and safety results from the SPYRAL HTN-ON MED proof-of-concept randomised trial. Lancet. 2018;391:2346–55. 10.1016/S0140-6736(18)30951-6. 29803589 10.1016/S0140-6736(18)30951-6

[CR42] Kario K, et al. Sufficient and persistent blood pressure reduction in the final long-term results from SYMPLICITY HTN-Japan - safety and efficacy of renal denervation at 3 years. Circ J. 2019;83:622–9. 10.1253/circj.CJ-18-1018. 30760655 10.1253/circj.CJ-18-1018

[CR43] Kario K, et al. Catheter-based ultrasound renal denervation in patients with resistant hypertension: the randomized, controlled REQUIRE trial. Hypertens Res. 2022;45:221–31. 10.1038/s41440-021-00754-7. 34654905 10.1038/s41440-021-00754-7 PMC8766280

[CR44] Katzman PL, Henningsen NC, Fagher B, Thulin T, Hulthén UL. Renal and endocrine effects of long-term converting enzyme inhibition as compared with calcium antagonism in essential hypertension. J Cardiovasc Pharmacol. 1990;15:360–4. 10.1097/00005344-199003000-00003. 1691357 10.1097/00005344-199003000-00003

[CR45] Kjeldsen SE, Narkiewicz K, Oparil S, Hedner T. Renal denervation in treatment-resistant hypertension - Oslo RDN, Symplicity HTN-3 and INSPiRED randomized trials. Blood Press. 2014;23:135–7. 10.3109/08037051.2014.916896. 24842262 10.3109/08037051.2014.916896

[CR46] Krum H, et al. Percutaneous renal denervation in patients with treatment-resistant hypertension: final 3-year report of the Symplicity HTN-1 study. Lancet. 2014;383:622–9. 10.1016/S0140-6736(13)62192-3. 24210779 10.1016/S0140-6736(13)62192-3

[CR47] Lambertucci L, et al. Trandolapril, but not verapamil nor their association, restores the physiological renal hemodynamic response to adrenergic activation in essential hypertension. Transl Res. 2011;157:348–56. 10.1016/j.trsl.2010.12.016. 21575919 10.1016/j.trsl.2010.12.016

[CR48] Lang CC, Choy AM, Balfour DJ, Struthers AD. Prazosin attenuates the natriuretic response to atrial natriuretic factor in man. Kidney Int. 1992;42:433–41. 10.1038/ki.1992.306. 1405327 10.1038/ki.1992.306

[CR49] Lang CC, Rahman AR, Balfour DJ, Struthers AD. Enalapril blunts the antinatriuretic effect of circulating noradrenaline in man. J Hypertens. 1993;11:565–71. 10.1097/00004872-199305000-00013. 8390529 10.1097/00004872-199305000-00013

[CR50] Lohmeier TE, Hall JE. Device-based neuromodulation for resistant hypertension therapy. Circ Res. 2019;124:1071–93. 10.1161/CIRCRESAHA.118.313221. 30920919 10.1161/CIRCRESAHA.118.313221 PMC6442942

[CR51] Lurz P, Fengler K. Lessons learned from RADIOSOUND-HTN: different technologies and techniques for catheter-based renal denervation and their effect on blood pressure. Interv Cardiol. 2019;14:102–6. 10.15420/icr.2019.03.R1. 31178937 10.15420/icr.2019.03.R1 PMC6545992

[CR52] Lurz P, et al. Changes in stroke volume after renal denervation: insight from cardiac magnetic resonance imaging. Hypertension. 2020;75:707–13. 10.1161/HYPERTENSIONAHA.119.14310. 32008429 10.1161/HYPERTENSIONAHA.119.14310

[CR53] Mathias CJ. Role of sympathetic efferent nerves in blood pressure regulation and in hypertension. Hypertension. 1991;18:III22-30. 10.1161/01.hyp.18.5_suppl.iii22. 1937684 10.1161/01.hyp.18.5_suppl.iii22

[CR54] McCorry LK. Physiology of the autonomic nervous system. Am J Pharm Educ. 2007;71:78. 10.5688/aj710478. 17786266 10.5688/aj710478 PMC1959222

[CR55] Memar K, et al. Low- and high-frequency spinal cord stimulation and arterial blood pressure in patients with chronic pain and hypertension: a retrospective study. Clin Auton Res. 2023a;33:443–9. 10.1007/s10286-023-00947-9. 37171770 10.1007/s10286-023-00947-9 PMC12077405

[CR56] Memar K, et al. Low- and high- frequency spinal cord stimulation and arterial blood pressure in patients with chronic pain and hypertension: a retrospective study. Clin Auton Res. 2023b;33:443–9. 10.1007/s10286-023-00947-9. 37171770 10.1007/s10286-023-00947-9 PMC12077405

[CR57] Obi MF, et al. The implementation of renal denervation in the management of resistant hypertension despite use of multitherapy antihypertensives at maximally tolerated doses: a contemporary literature review. Cureus. 2023;15:e41598. 10.7759/cureus.41598. 37559838 10.7759/cureus.41598 PMC10409301

[CR58] Oliveras A, et al. Spironolactone versus sympathetic renal denervation to treat true resistant hypertension: results from the DENERVHTA study - a randomized controlled trial. J Hypertens. 2016;34:1863–71. 10.1097/HJH.0000000000001025. 27327441 10.1097/HJH.0000000000001025 PMC4972478

[CR59] Papademetriou V, Doumas M, Tsioufis K. Renal sympathetic denervation for the treatment of difficult-to-control or resistant hypertension. Int J Hypertens. 2011a;2011:196518. 10.4061/2011/196518. 21629864 10.4061/2011/196518 PMC3095896

[CR60] Papademetriou V, Tsioufis K, Gradman A, Punzi H. Difficult-to-Treat or Resistant Hypertension: Etiology, Pathophysiology, and Innovative Therapies. Int J Hypertens. 2011b;2011:438198. 10.4061/2011/438198. 21822477 10.4061/2011/438198 PMC3124360

[CR61] Pathak A, et al. Alcohol-mediated renal denervation in patients with hypertension in the absence of antihypertensive medications. EuroIntervention. 2023;19:602–11. 10.4244/EIJ-D-23-00088. 37427416 10.4244/EIJ-D-23-00088 PMC10493775

[CR62] Ram CV, Kumar AS. Renal denervation therapy for resistant hypertension: a clinical update. J Hum Hypertens. 2014;28:699–704. 10.1038/jhh.2014.6. 24599151 10.1038/jhh.2014.6

[CR63] Ram CV, et al. Renal denervation therapy for hypertension: truths and half-truths: Renal denervation therapy for hypertension. AsiaIntervention. 2021;7:62–8. 10.4244/AIJ-D-21-00013. 34913005 10.4244/AIJ-D-21-00013 PMC8657029

[CR64] Ram CVS. Renal denervation therapy for hypertension: all that glitters is not gold. Eur Heart J. 2022;43:2177–8. 10.1093/eurheartj/ehac065. 35171252 10.1093/eurheartj/ehac065

[CR65] Raman VK, Tsioufis C, Doumas M, Papademetriou V. Renal denervation therapy for drug-resistant hypertension: does it still work? Curr Treat Options Cardiovasc Med. 2017;19:39. 10.1007/s11936-017-0536-4. 28455809 10.1007/s11936-017-0536-4

[CR66] Ranard LS, Swaminathan RV. Renal Artery Denervation for Hypertension. Curr Treat Options Cardiovasc Med. 2019;21:7. 10.1007/s11936-019-0715-6. 30762119 10.1007/s11936-019-0715-6

[CR67] Reid JL. Alpha-adrenergic receptors and blood pressure control. Am J Cardiol. 1986;57:6E-12E. 10.1016/0002-9149(86)90716-2. 2869681 10.1016/0002-9149(86)90716-2

[CR68] Rosa J, et al. Randomized comparison of renal denervation versus intensified pharmacotherapy including spironolactone in true-resistant hypertension: six-month results from the Prague-15 study. Hypertension. 2015;65:407–13. 10.1161/HYPERTENSIONAHA.114.04019. 25421981 10.1161/HYPERTENSIONAHA.114.04019

[CR69] Rosa J, et al. Role of adding spironolactone and renal denervation in true resistant hypertension: one-year outcomes of randomized PRAGUE-15 Study. Hypertension. 2016;67:397–403. 10.1161/HYPERTENSIONAHA.115.06526. 26693818 10.1161/HYPERTENSIONAHA.115.06526

[CR70] Rosa J, et al. Renal denervation in comparison with intensified pharmacotherapy in true resistant hypertension: 2-year outcomes of randomized PRAGUE-15 study. J Hypertens. 2017;35:1093–9. 10.1097/HJH.0000000000001257. 28118281 10.1097/HJH.0000000000001257

[CR71] Sarwar M. S, Islam M. S, Al Baker S. M, Hasnat A. Resistant hypertension: underlying causes and treatment. Drug Res (Stuttg). 2013;63:217–23. 10.1055/s-0033-1337930. 23526242 10.1055/s-0033-1337930

[CR72] Scheffers IJ, et al. Novel baroreflex activation therapy in resistant hypertension: results of a European multi-center feasibility study. J Am Coll Cardiol. 2010;56:1254–8. 10.1016/j.jacc.2010.03.089. 20883933 10.1016/j.jacc.2010.03.089

[CR73] Schultz DM, et al. Acute cardiovascular effects of epidural spinal cord stimulation. Pain Physician. 2007;10:677–85. 17876365

[CR74] Schultz DM, Zhou X, Singal A, Musley S. Cardiovascular effects of spinal cord stimulation in hypertensive patients. Pain Physician. 2011;14:1–14. 21267037

[CR75] Schmieder RE, et al. Adherence to antihypertensive medication in treatment-resistant hypertension undergoing renal denervation. J Am Heart Assoc. 2016;5:e002343. 10.1161/JAHA.115.002343. 26873693 10.1161/JAHA.115.002343 PMC4802436

[CR76] Schmieder RE, et al. Phase II randomized sham-controlled study of renal denervation for individuals with uncontrolled hypertension - WAVE IV. J Hypertens. 2018;36:680–9. 10.1097/HJH.0000000000001584. 29035942 10.1097/HJH.0000000000001584

[CR77] Schneider S, et al. Impact of sympathetic renal denervation: a randomized study in patients after renal transplantation (ISAR-denerve). Nephrol Dial Transplant. 2015;30:1928–36. 10.1093/ndt/gfv311. 26333545 10.1093/ndt/gfv311

[CR78] Squair JW, et al. Neuroprosthetic baroreflex controls haemodynamics after spinal cord injury. Nature. 2021;590:308–14. 10.1038/s41586-020-03180-w. 33505019 10.1038/s41586-020-03180-w

[CR79] Sverrisdottir YB, et al. Human dorsal root ganglion stimulation reduces sympathetic outflow and long-term blood pressure. JACC Basic Transl Sci. 2020;5:973–85. 10.1016/j.jacbts.2020.07.010. 33145461 10.1016/j.jacbts.2020.07.010 PMC7591825

[CR80] Townsend RR, et al. Catheter-based renal denervation in patients with uncontrolled hypertension in the absence of antihypertensive medications (SPYRAL HTN-OFF MED): a randomised, sham-controlled, proof-of-concept trial. Lancet. 2017;390:2160–70. 10.1016/S0140-6736(17)32281-X. 28859944 10.1016/S0140-6736(17)32281-X

[CR81] Undrum Bergland O, et al. Changes in sympathetic nervous system activity after renal denervation: results from the randomised Oslo RDN study. Blood Press. 2021;30:154–64. 10.1080/08037051.2020.1868286. 33399016 10.1080/08037051.2020.1868286

[CR82] Van Beusecum J, Inscho EW. Regulation of renal function and blood pressure control by P2 purinoceptors in the kidney. Curr Opin Pharmacol. 2015;21:82–8. 10.1016/j.coph.2015.01.003. 25616035 10.1016/j.coph.2015.01.003 PMC5515225

[CR83] van der Stricht J. Indications for lumbar sympathectomy. J Cardiovasc Surg (Torino). 1979;20:339–40. 447772

[CR84] van Kleef M, Bates MC, Spiering W. Endovascular baroreflex amplification for resistant hypertension. Curr Hypertens Rep. 2018;20:46. 10.1007/s11906-018-0840-8. 29744599 10.1007/s11906-018-0840-8 PMC5942348

[CR85] van Kleef M, et al. Endovascular baroreflex amplification and the effect on sympathetic nerve activity in patients with resistant hypertension: a proof-of-principle study. PLoS ONE. 2021;16:e0259826. 10.1371/journal.pone.0259826. 34784359 10.1371/journal.pone.0259826 PMC8594823

[CR86] van Kleef M, et al. Treatment of resistant hypertension with endovascular baroreflex amplification: 3-year results from the CALM-FIM study. JACC Cardiovasc Interv. 2022;15:321–32. 10.1016/j.jcin.2021.12.015. 35144789 10.1016/j.jcin.2021.12.015

[CR87] van Lieshout JJ, Wieling W, Wesseling KH, Endert E, Karemaker JM. Orthostatic hypotension caused by sympathectomies performed for hyperhidrosis. Neth J Med. 1990;36:53–7. 2314521

[CR88] Voora R, Hinderliter AL. Modulation of Sympathetic Overactivity to Treat Resistant Hypertension. Curr Hypertens Rep. 2018;20:92. 10.1007/s11906-018-0893-8. 30194545 10.1007/s11906-018-0893-8

[CR89] Welch V, et al. PRISMA-Equity 2012 extension: reporting guidelines for systematic reviews with a focus on health equity. PLoS Med. 2012;9:e1001333. 10.1371/journal.pmed.1001333. 23222917 10.1371/journal.pmed.1001333 PMC3484052

[CR90] Zhang J, Zhou S, Xu G. Carotid Baroreceptor Stimulation: A Potential Solution for Resistant Hypertension. Interv Neurol. 2014;2:118–22. 10.1159/000357167. 25187787 10.1159/000357167 PMC4062316

